# Identification and Characterization of Defects in Glass Fiber Reinforced Plastic by Refining the Guided Lamb Waves

**DOI:** 10.3390/ma11071173

**Published:** 2018-07-09

**Authors:** Kumar Anubhav Tiwari, Renaldas Raisutis

**Affiliations:** 1 Prof. K. Barsauskas Ultrasound Research Institute, Kaunas University of Technology, K. Baršausko St. 59, LT-51423 Kaunas, Lithuania; renaldas.raisutis@ktu.lt; 2 Department of Electrical Power Systems, Faculty of Electrical and Electronics Engineering, Kaunas University of Technology, Studentu St. 48, LT-51367 Kaunas, Lithuania

**Keywords:** glass fiber reinforced plastic, non-destructive testing, ultrasonic, guided wave, macro-fiber composite, wavelet transform, amplitude detection, two-dimensional fast Fourier transform, Hilbert transform, variational mode decomposition

## Abstract

In this paper, the disbond-type defect presented on glass fiber reinforced plastic material is analyzed by refining the guided Lamb wave signals. A segment of wind turbine blade is considered as a test sample. The low-frequency ultrasonic measurement system is used for the non-destructive testing of the test sample using guided waves. The P-1 type macro-fiber composite transducer as a transmitter and contact-type piezoceramic transducer as a receiver are used for the testing of a sample. The disbond type defect having a diameter of 81 mm is detected from the experimental results. To improve the accuracy in locating and sizing the defects and estimation of the time of flight and phase velocity of ultrasonic guided waves in defective region, signal processing algorithm is developed by utilizing the promising properties of various ultrasonic signal processing techniques such as wavelet transform, amplitude detection, two-dimensional Fast-Fourier transform, Hilbert transform and variational mode decomposition. The discrete wavelet transform is used to denoise the guided wave signals and then, the size and location of defects are estimated by amplitude detection. The reflected wave signals from the opposite edge of the sample are removed by applying the two-dimensional Fast-Fourier transform to the experimental B-scan signal. Afterwards, variational mode decomposition and Hilbert transform are used for the phase velocity and time-delay estimation by comparing the instantaneous amplitudes of the defective and defect-free signal. The validation and the demonstration of reproducibility of the algorithm is performed by extracting the features of a 51 mm defect from another experimental B-scan.

## 1. Introduction

The composite materials such as glass fiber reinforced plastic (GFRP) and carbon fiber reinforced plastic (CFRP) are widely used for the construction of various complex structures [1,2,3]. However, they are most commonly used to manufacture the components operating under varying load applications such as the blades of wind turbines and aircraft [4]. There are many advantages associated with composites materials as compared to metals. They have lighter weight, lower density, higher stiffness and higher compressive and tensile strengths in comparison to their metallic counterparts [5]. Despite the favorable features, the various kinds of manufacturing or in-service defects may exist in composite materials. The manufacturing defects may occur due to defective fibers, misalignment of ply, delamination, and flaws, whereas cracks and disbonds on structure, damages due to ageing and moisture can be formed *in-service* [6,7].

Many non-destructive testing (NDT) techniques have been developed for the estimation of location and size of defects. However, the ability to cover a long distance, high sensitivity to the defective regions and requirement of only a little training for operating the equipment are the distinguished advantages of ultrasonic guide wave (GW) testing in comparison to other testing methods. Hence, GW testing is the most optimistic approach for the identification of defects in composite materials [8,9]. The researchers have successfully inspected the composite structures for the identification of different kind of defects using ultrasonic GW [10,11,12,13,14,15]. Various types of guided waves (GWs) are available, such as Rayleigh or surface waves and Lamb waves which can propagate in bounded media. Lamb wave is a special kind of ultrasonic GW, which has more sensitivity to the internal or surface defects over the thickness of the material [16,17,18]. One of the challenges in GW testing of multi-layered composite structures is the high attenuation of guided wave modes as it passes through the structure [19]. Many factors such as excitation frequency of a transducer, type of material, geometry of the structure, operating conditions and direction of propagating GW modes may affect the behavior of received signals [8,9,20]. During the interaction of GW signals with multi-layered complex structures, the wave phenomenon such as scattering, reflection, mode conversion, refraction or attenuation may occur. Hence, the estimation of defect size and location becomes very difficult from the received GW signals. One of the most common methods used in structural health monitoring systems (SHM) is the comparative analysis of reference and current states for identifying the defects or damages [21,22]. The reference signal is acquired in the defect-free region. The information of the defects can be extracted by the algebraic difference of the reference and current time signals which eliminates the boundary reflections [23,24]. However, this method is used only to detect the defects as it provides no information about the location and size of defects rather than the marginal presence of defects. Therefore, signal processing techniques for the refinement of received GW signals are required for the estimation of location and size of the defects.

The objective of this research is to locate, size and characterize the disbond type defect presented on a GFRP sample by applying signal processing techniques to the GW signals. The sample has been a segment of wind turbine blade (WTB). A signal processing algorithm based on discrete wavelet transform (DWT), amplitude detection, two-dimensional Fast-Fourier transform (2D-FFT), variational mode decomposition (VMD) and Hilbert transform (HT) is developed to increase the accuracy in the defect analysis. The disbond-type defect having 81 mm diameter (D81) was located on the main spar of the sample. The DWT removes the non-stationary noise. The amplitude detection is applied to the wavelet-denoised signals for the estimation of defect size and location. The reflected signals are suppressed from the experimental B-scan by 2D-FFT. The VMD removes the correlated noise by decomposing the selective A-scan signals of defect-free and defective regions into monocomponent signals. Finally, the HT is applied on the reconstructed signals after VMD analysis to calculate the time-delays and phase velocities of GW signals in the defect-free and defective regions by comparing the instantaneous amplitudes. The novelty in the research is the adaptation of these signal processing methods to characterize the defect parameters from a single B-scan. The combination of 2D-FFT with VMD could be an effective approach if the 2D-FFT itself cannot estimate the correct values of phase velocities and time of arrival of the propagating GW modes. The developed signal processing approach is also validated in the case of 51 mm defect.

The paper is organized as follows: Signal processing techniques to process the GW signals are introduced in Section 2. Experimental investigation of GFRP sample using guided Lamb waves including the description of low-frequency (LF) ultrasonic system, GFRP sample and transducers are presented in Section 3. Signal processing algorithm to improve the accuracy in the defect analysis is described in Section 4. Application of signal processing algorithm on experimental signals is demonstrated in Section 5. In Section 6, the signal processing algorithm is validated in the case of another experimental investigation of a 51 mm defect by contact-type transducers. The limitations and issues of this approach are discussed in Section 7. Section 8 summarizes the final conclusions and future scope of this research.

## 2. Overview of GW Signal Processing Techniques

As discussed in Section 1, it is very difficult to estimate the size and location of defects from the received GW signals during experiments. Signal processing is necessary for denoising and analyzing the variation in one or more of the signal parameters such as time of flight, amplitude, frequency, etc. This facilitates extracting the information about defects presented in the structure [25,26]. The signal processing techniques for the post-processing of GW signals include DWT, HT, amplitude detection, cross-correlation, 2D-FFT, Hilbert–Huang transform (HHT), split-spectrum processing (SSP), short-time Fourier transform (STFT) and mode decomposition techniques such as empirical mode decomposition (EMD), ensemble empirical mode decomposition (EEMD) and VMD [27,28,29,30,31,32,33,34,35].

In this work, features of DWT, amplitude detection, 2D-FFT, VMD and HT are used for the estimation and characterization of defects. A brief overview of these techniques is as follows.

### 2.1. DWT and Amplitude Detection

The Wavelet processing is widely used to reduce the structural and grain noise from the structure in ultrasonic NDT of the composite structures [27,36,37]. It increases the signal-to-noise ratio and potential capabilities for the estimation of size and location of defects. The nonstationary signals can be comprehensively processed in both time-domain and frequency-domain by using the DWT [27]. In this technique, a time-domain signal is decomposed into elementary signals. These elementary signals are called wavelets. The correlation between original signal and wavelets is wavelet transform. The available wavelet functions are called mother wavelet, which is used to generate the daughter wavelets by using the dilation and shifting process.

In the process of wavelet noising, the information of the signal is preserved while reducing the signal components of smaller amplitudes without consideration of frequencies. The wavelet coefficients are altered or discarded for the noise removal using DWT [38,39]. The best way for the manipulation of wavelet coefficients in the case of correlated noise is a soft-threshold technique with universal threshold [40]. In this research, Daubechies (db) mother wavelet is used due to its high energy preserving capabilities as compared to other wavelet families [41]. After denoising using DWT, an amplitude detection technique is generally applied with an appropriate decision level to distinguish between the amplitude variations in defect-free and defective regions [26]. In this way, approximated size and location of the defects can be predicted. The basic steps with mathematical expressions are described as follows:

- After selecting a wavelet-type, the DWT utilizes low-pass filtering for scaling function and high-pass filtering for wavelet function. If a signal *x*[*n*] is processed by a half-band high-pass filter (HPF) and a low-pass filter (LPF) having filter functions of $h_{hp}\left[n\right]$ and $h_{lp}\left[n\right]$ respectively, half of the samples are eliminated by Nyquist criterion and, therefore, the level-1 decomposition is initiated. The filter responses can be expressed by [42,43]:
  (1) $$y_{h}\left[k\right]=\underset{n}{\sum }x\left[n\right]\cdot h_{hp}\left[2k-n\right]$$
  (2) $$y_{l}\left[k\right]=\underset{n}{\sum }x\left[n\right]\cdot h_{lp}\left[2k-n\right]$$
  where $y_{h}\left[k\right]\;$ and $y_{l}\left[k\right]\;$ are the response of the HPF and LPF, respectively (achieved after sub-sampling by 2).

The process is repeated for further decomposition.

- The decomposition level *k* lies between 1 and the maximum level of decomposition (*M*).
  (3) $$M=log_{2}N;\;N\;\mathrm{is}\;\mathrm{the}\;\mathrm{length}\;\mathrm{of}\;\mathrm{signal}\;x[n].$$
- The noise threshold for the detailed components using universal threshold is given by [44]:
  (4) $$\lambda ={\sigma_{j}}^{\bar{noise}}\sqrt{2\log\left(N\right)}$$
  where ${\sigma_{j}}^{\bar{noise}}$ is estimated noise level.
- After denoising of detailed components, the inverse-DWT (IDWT) is applied to *k* detail components and *k*th approximation component to generate the denoised signal.

### 2.2. 2D-FFT

To convert the time–distance measurements into the frequency–phase velocity domain, 2D fast Fourier transform (FFT) is widely used. The 2D-FFT method states that the wave propagation along the plate can be expressed by the transfer function (*H*) of scanning distance and arrival time and can be transformed into the wavenumber (*k*) and frequency space [35,45]:

(5) $$H\left(k,\omega \right)=\overset{+\infty }{\underset{-\infty }{\int }}\overset{+\infty }{\underset{-\infty }{\int }}u\left(x,t\right)\cdot e^{-j\left(kx+\omega t\right)}dxdt$$

where *x* is the scanning distance, *t* is the arrival time, *k* is the wavenumber, *ω* is the angular frequency (*ω* = 2 · *π* · *f*) and *f* is the frequency.

Since the phase velocity is the ratio of angular frequency and wavenumber, the phase velocity dispersion curve can be reconstructed. However, there is a possibility that the dominant modes at excitation frequency cannot be estimated correctly from the phase dispersive characteristics due to side and background reflections and mode conversions. In that case, the B-scan must be reconstructed separately for the direct and reflected from the opposite edge of the sample waves by using 2D-FFT.

### 2.3. Mode Decomposition Technique: VMD

Mode decomposition techniques are used to decompose the nonlinear and nonstationary multicomponent signals into principal components which are also called as intrinsic mode function (IMF) or “modes”. The noisy modes can be eliminated from the reconstructed signal. The EMD decomposition technique developed by Huang et al. [30] is based on the detection of local minima/maxima of the signal in a recursive manner and applying the interpolation of extrema for estimating the lower or upper envelopes. Thereafter, high-frequency components are eliminated by filtering out the mean of envelopes or mode. The serious limitations associated with EMD are the dependence of decomposition procedure on extrema calculation, interpolation of extrema into envelopes, and the imposition of stopping criteria. Some limitations of EMD were overcome by EEMD decomposition process. The end effect problem due to spline fitting process to estimate envelope and mode mixing problem due to same oscillations in different IMFs or multiple oscillations in same IMF was removed in EEMD process [31].

The VMD developed by Dragomiretskiy and Zosso [34] is based on application and conversion of Wiener filter into the different bands. As the amplitudes and frequency are not considered in the process, all kind of oscillations can be separated by VMD. Moreover, the inverse problem associated with EMD and EEMD is overcome by decomposing modes using least squares. Therefore, a definite bandwidth is assigned to each mode. Although VMD is based on EMD, it is more robust to noise associated with GWs in composite materials.

To achieve bandwidth-constrained mode, the following procedure is performed [34]:

- The unilateral frequency spectrum is obtained for each mode by using HT.
- The exponential tuned to center frequency is added to the frequency spectrum. In this way, the frequency spectrum of each mode is shifted to baseband.
- Finally, by means of H1 Gaussian smoothness of the demodulated signal, the bandwidth is calculated.

Hence, the resulting variational problem can be expressed as [34]:

(6) $$\underset{u_{k},\omega_{k}}{\min}{\left\lfloor \overset{K}{\underset{k=1}{\sum }}\|\partial_{t}\left(\left[\delta \left(t\right)+\frac{j}{\pi t}\right]*u_{k}\left(t\right)\right)e^{-i\omega_{k}t}\|\right\rfloor }_{2}^{2}$$

where *u_k_* (*k* = 1, 2…. *K*) is set of all modes for a real-valued signal (x); *ω_k_* is a set of their center frequencies; *δ* is Dirac distribution; and (*) denotes convolution.

(7) $$\overset{K}{\underset{k=1}{\sum }}u_{k}=x$$

The reconstruction constraint is addressed by combining the properties of Lagrangian multipliers (λ) and quadratic penalty, and called augmented Lagrangian [46,47].

### 2.4. HT and Instantaneous Characteristics

The instantaneous amplitude and instantaneous frequency of a band-bounded and real-valued signal are estimated by HT [48]. For a real-valued signal *x*(*t*), the HT *X_h_*(*t*) is given as:

(8) $$X_{h}\left(t\right)=\frac{1}{\pi }\int_{-\infty }^{\infty }\frac{x\left(u\right)}{t-u}du$$

The analytical signal *x_a_(t)* and its relation with real-valued signal *x(t)* can be expressed by:

(9) $$x_{a}\left(t\right)=x\left(t\right)+iX_{h}\left(t\right)=A_{i}\left(t\right).e^{i\varphi \left(t\right)}$$

where *A*_*i*_(*t*) and φ(t) are instantaneous amplitude and instantaneous phase, respectively, of *x(t)*.

The instantaneous amplitude *A_i_*(*t*) and instantaneous frequency *f_i_*(*t*) can be expressed as follows:

(10) $$A_{i}\left(t\right)=\left|x\left(t\right)+iX_{h}\left(t\right)\right|$$

(11) $$f_{i}\left(t\right)=\frac{1}{2\pi }\frac{d\varphi \left(t\right)}{dt}$$

In the case of GW signals, the time delay estimation is possible by comparing the instantaneous amplitudes of defect-free and defective signals. The instantaneous frequencies of the defect-free and defective signals do not provide information for the other parameters, but the variation in frequency for GW signals can be compared in defective and defect-free regions.

It should be noted that GW signals must be converted into the monocomponent signals to estimate the instantaneous characteristics. Moreover, there is no authenticity of analytical signals for the application of HT, if harmonics are not decomposed properly [49]. Hence, mode decomposition procedure must be applied before proceeding the HT.

## 3. Experimental Analysis of GFRP Sample

### 3.1. Sample and Devices

**Figure 1:**
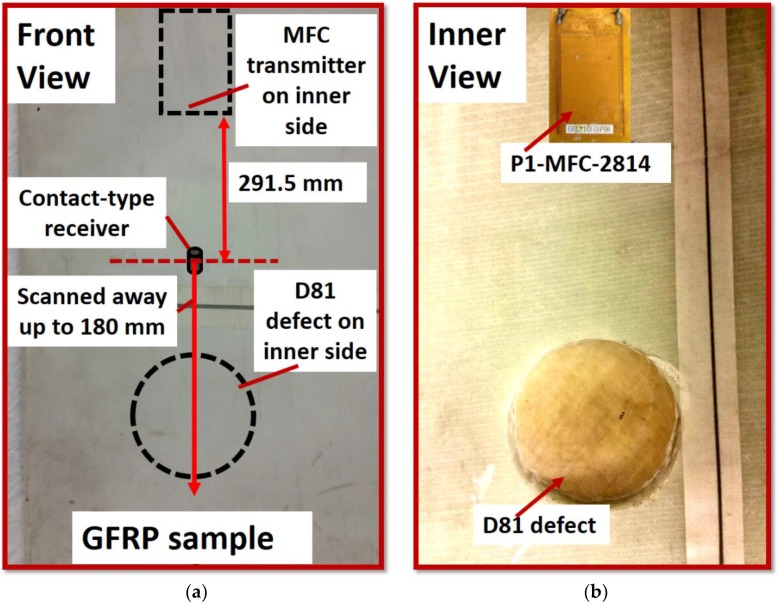

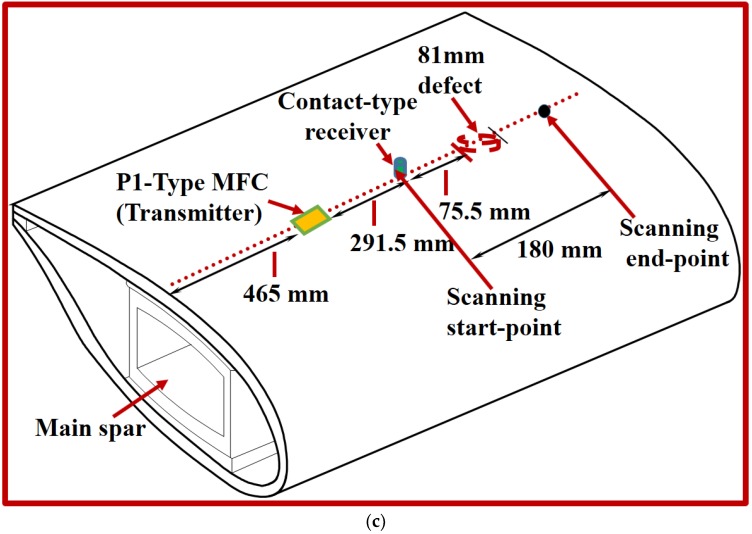
GFRP sample and arrangement of transducers: (**a**) front view of the sample where scanning is performed; (**b**) inner view of sample where defect of 81 mm diameter is present and MFC transducer is glued; and (**c**) schematic showing the segment of WTB, arrangement and position of transducers and location of defect.

The GFRP sample (Figure 1a,b) was a segment of WTB. The disbond-type artificial defect with diameter 81 mm was located on the main spar of the sample. The artificial defect was created by the mechanical machining (milling process). The thickness of the sample varied between 20 and 23 mm in the defect-free region. On another hand, the thickness in the defective region was between 3 and 4 mm. The inner photo view of the sample showing the D81 defect is presented in Figure 1b. It should be noted that defect was not visible from the surface side during the experimental scanning procedure.

The P1-type macro-fiber composite (P1-MFC-2814) [50] transducer is used as a GW transmitter which is widely used for NDT and SHM of composite structures. Due to its small size, lightweight, high reliability and durability, the macro-fiber composite (MFC) transducers can be easily embedded with the long and composite structures [51]. One of the best features of MFC transducers is that they are efficient transmitters and receivers of the fundamental asymmetric (the A0) and symmetric (the S0) modes of guided Lamb waves [52,53,54]. As guided Lamb waves can propagate up to the long distance through the material with less attenuation, it is used to extract the information about the subsurface defects and delaminations [55,56]. The MFC transducer was glued on the inner side of the sample (Figure 1b). Although P1-MFC produces the S0 mode in a more dominant manner as compared to the A0 mode, the interested GW mode for the analysis of defects also depends on the type of receiving transducer.

The point-type piezoceramic transducer (Figure 1a) is used as a receiver to record the GW signals during the experimental scanning of a sample. The 6 dB bandwidth of a receiving transducer was up to 300 kHz [57]. The center frequency is 190 kHz. The −10 dB bandwidth was from 35 kHz to 640 kHz which covers the excitation frequency of 43 kHz [57]. To proceed with the contact-type ultrasonic testing by scanning away from this transducer, a conical-shaped protection layer of 2 mm diameter was provided at the bottom of the transducer. The schematic of sample and placement of MFC transmitter and contact-type receiver is shown in Figure 1c. The initial position of the contact-type receiver was at 291.5 mm from the MFC transmitter and it was scanned away up to 180 mm passing through the defective region. The initial end-to-end distance between the receiver and the D81 defect was 75.5 mm.

The contact type receiver was attached to the mechanical scanner which was connected to the low-frequency (LF) ultrasonic system developed by the Kaunas University of Technology, Kaunas, Lithuania. The parametric specifications of LF ultrasonic system are presented in Table 1 [32,58,59].

### 3.2. GW Testing of Sample

To test the sample using guided Lamb waves, P1-MFC transducer glued on the inner side of the sample was excited with 43 kHz, three-period excitation signal. The point-type piezoceramic transducer on the outer side of sample was scanned away up to 180 mm to record the GW signals (A-scans) with a step of 0.2 mm. The sampling frequency was equal to 100 MHz. The excitation frequency of 43 kHz was selected because it was closer to the resonant frequency of the MFC transducer [54]. During the scanning procedure, a coupling is required to maintain the acoustic contact between the piezoceramic receiver and the surface of the sample. Glycerol was used for this purpose. The LF ultrasonic system was used for the experimental analysis. The defects were not visually visible from the outer surface during the investigation. The low-frequency (LF) ultrasonic system and all components except MFC transducer used in the experiment were developed by Ultrasound Research Institute of Kaunas University of Technology.

**Figure 2:**
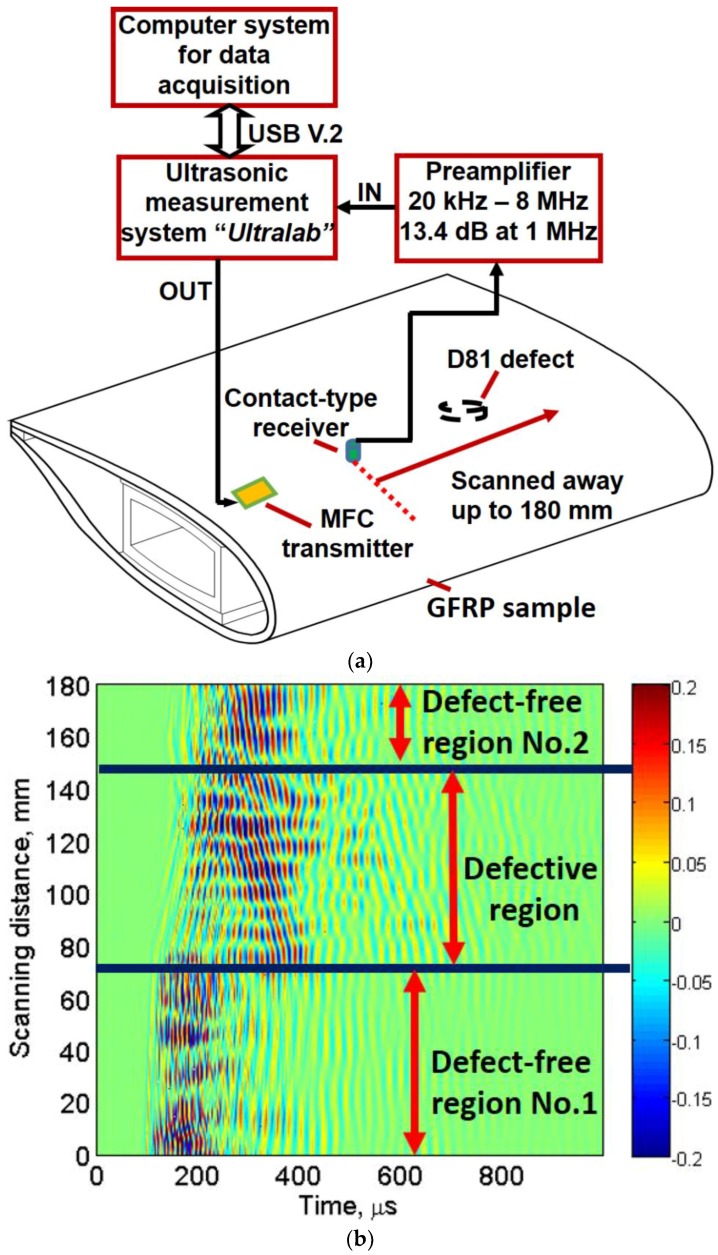
GW testing: (**a**) schematic of experimental investigation of the sample; and (**b**) acquired B-scan image from the linear scanning.

The location of transducers, D81 defect, characteristics of transducers and LF ultrasonic system is described in Section 3.1. The schematic of experimental analysis is shown in Figure 2a. The acquired B-scan image from the linear scanning is shown in Figure 2b along the distance (0–180 mm) and time (0–1000 µs). It can be clearly observed in Figure 2b that GWs are scattered and reflected in the part of B-scan. Thus, defect-free and defective regions can approximately be determined. However, the location, size and characteristics of the defect could not be determined. Hence, a signal processing approach was required for the analysis of defect from experimental B-scan. It should be noted that receiving point-type transducer operates in thickness mode, which is more sensitive to out-of-plane radiations. Although both A0 and S0 contain *out-of-plane* displacements, A0 has more dominant *out-of-plane* displacements as compared to the S0. Therefore, the analysis was based on the A0 mode in the registered B-scan image.

## 4. Signal Processing Algorithm

**Figure 3:**
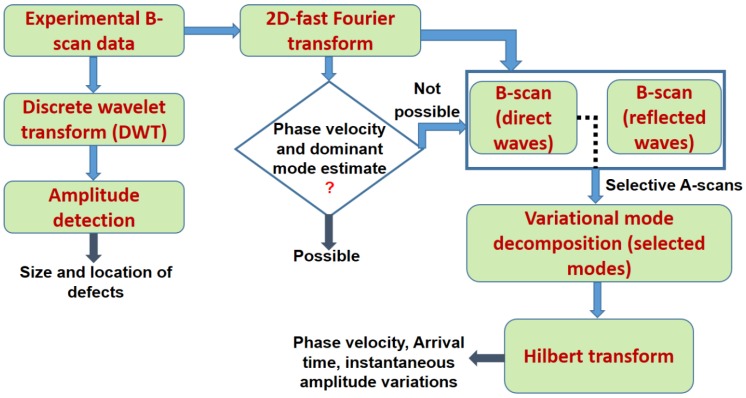
Signal processing algorithm for the extraction of defect parameters.

The defect characterization and estimation can be possible by signal processing of GW signals. For this purpose, a signal processing algorithm that uses the features of DWT, amplitude detection, 2D-FFT, HT and VMD was developed. The algorithm is presented in Figure 3. There are two data paths. One data path corresponds to the estimation of defect size and location, whereas the other data path is used for the estimation of phase velocities and time delays of the defect-free and defective signal. The description of the signal processing algorithm follows.

- The DWT is applied to all A-scan signals within the applied window to the experimental B-scan (Figure 2b) for signal denoising, as described in Section 2.1. The window must be selected in such a way so that full defective region and partial defect-free region No. 1 and defect-free region No. 2 is covered. The Daubechies (*db*) mother wavelet, as described in Section 2.1, is used for the signal decomposition.
- After wavelet denoising, the amplitude detection technique is used to plot the normalized amplitudes along the scanning distance (0 to 180 mm). The decision threshold of −3 dB is applied to estimate the size and location of the D81 defect.
- To calculate the phase velocities of dominant GW mode (the A0) in the defect-free and defective regions, 2D-FFT is applied to the B-scan signal as described in Section 2.2. If phase velocity estimation is not possible due to dispersion, scattering, reflection, mode conversion or superimposition, the B-scan for the direct waves and reflected waves should be reconstructed separately.
- Two A-scan signals each from the defective and defect-free regions over a fixed distance are selected to apply the VMD for the suppression of correlated noise and mode-mixing, as described in Section 2.3. The A-scan signals are reconstructed by selecting the appropriate IMFs.
- Finally, the HT is applied to A-scans for the estimation of variations in instantaneous amplitudes with time as explained in Section 2.4. By applying the −3 dB thresholds, the time of arrivals can be calculated and with the known distance, the phase velocities in the defect-free and defective regions can be calculated.

## 5. Application of Signal Processing Algorithm on GW Signals

The application of signal processing approach presented in Section 4 to the experimentally obtained GW signals is presented in this section.

### 5.1. Defect Estimation (Size and Location of D81 Defect) by DWT

In the next step, the DWT is applied to the experimental B-scan by using Daubechies (db) mother wavelet and soft-threshold process with a universal threshold as discussed in Section 2.1. To remove the grainy and nonstationary noise with maintaining the signal shape and information, the appropriate level of the wavelet family should be selected. The db8 level was selected for the decomposition of each A-scan signal of the windowed B-scan into eight levels.

The selection of the db8 level was based on the correlation between the decomposed and original signals [60] and described as follows.

- First, three A-scan signals at 120 mm, 60 mm and 170 mm were selected each from the defective, defect-free region No. 1 and defect-free region No. 2, respectively, of the B-scan signal.
- After applying the DWT with db2, db4, db8 and db16 levels, the detailed signals at eighth level were reconstructed for each of three selected A-scans.
- The correlation coefficient between the original A-scans and the detailed signals were estimated.

**Figure 4:**
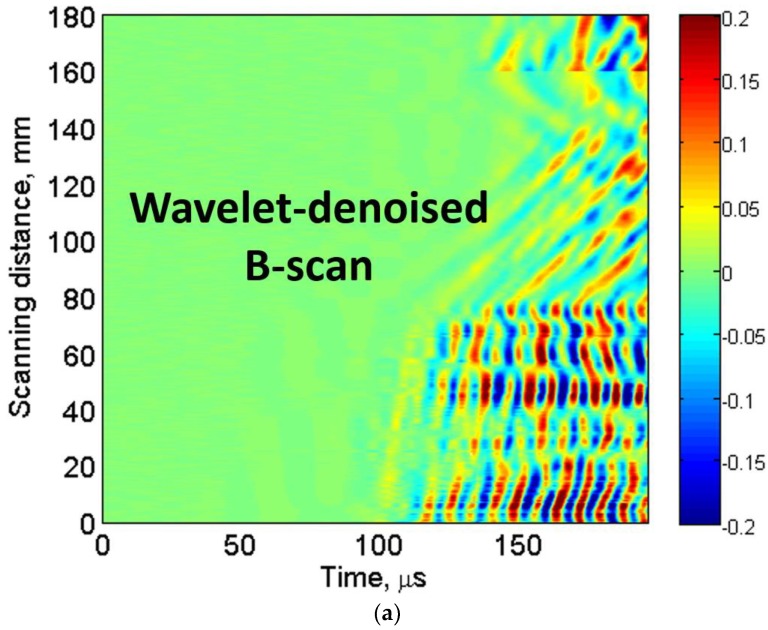

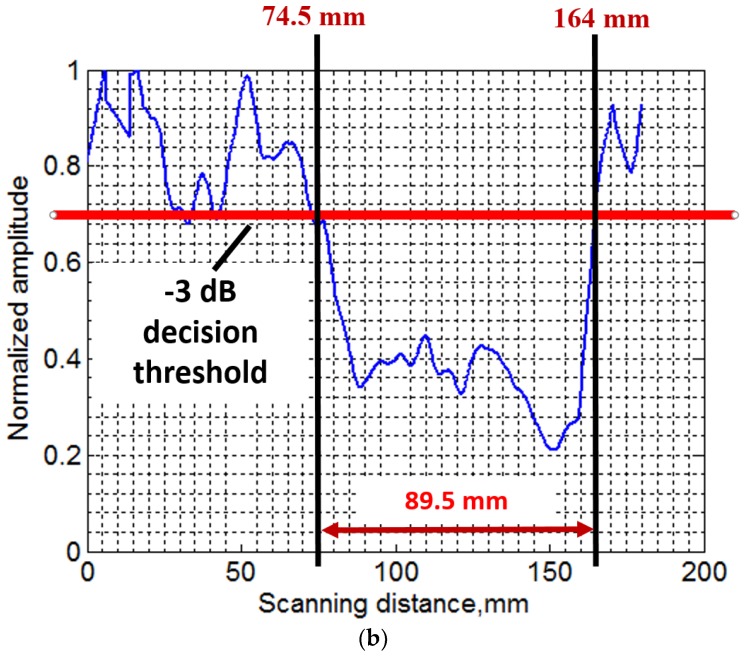
Defect estimation using DWT: wavelet denoised B-scan using db8 mother wavelet (**a**); and application of amplitude detection for estimating the size and location of 81 mm (D81) defect (**b**).

It was observed that detailed signals reconstructed with db8 achieved the highest correlation with their original A-scan signals in all three cases (defective, defect-free region No. 1 and defect-free region No. 2). Hence, db8 was used for the decomposition of signals. The wavelet-denoised B-scan within time (0–200 µs) is shown in Figure 4b. The defective region can be more clearly observed in wavelet denoised B-scan (Figure 4a) as compared to the experimental B-scan (Figure 2b). In the next step, the peak-to-peak amplitude of each A-scan of denoised B-scan image was calculated. The amplitude detection technique was then applied to present the variations of normalized amplitudes with respect to the scanned distance as shown in Figure 4b.

In our previous works, it was observed that amplitude of the out-of-plane component of propagating waves reduced significantly in the defective region due to one of the possible wave phenomenon of dispersion, scattering, reflection, mode conversion, etc. [13,61,62]. As we used the contact-type transducers operating in the thickness mode, amplitude detection technique was an efficient way to locate and size the defect. The decision threshold level of −3 dB (0.707) was applied for the estimation of size and location of the D81 defect. The D81 defect is clearly observed in Figure 4c. In Figure 4b, the estimated value of the location of defect from the receiving transducer is 74.5 mm (true value is 75.5 mm) with the measurement error of 1.3%, while the size of D81 defect is estimated as 89.5 mm (true value is 81 mm) with the measurement error of 10.5%.

A common technique to estimate the defect size and location in the structure in ultrasonic NDT is the two-dimensional scanning of the structure (ultrasonic C-scan) [63,64,65]. However, it is very time-consuming, even for the moderate objects despite the limited requirement of signal processing. Moreover, in the case of large structures with one side accessibility or structures with complex geometry, the C-scan becomes an impractical approach [66]. Therefore, the obtained results from a single B-scan with the appropriate signal processing show the significant accuracy in measurement of defect size and location with an error less than 11%. However, the B-scan imaging is useful only if the scanning transducers pass through the middle part of the defects.

### 5.2. Defect Characterization (Estimation of Time Delay and Phase Velocity) by 2D-FFT, VMD and HT

As discussed in Section 4, the features of 2D-FFT, VMD and HT were used for defect characterization in a sequential order.

#### 5.2.1. Dispersion Curves Using the Semi-Analytical Finite Element (SAFE) Method

**Figure 5:**
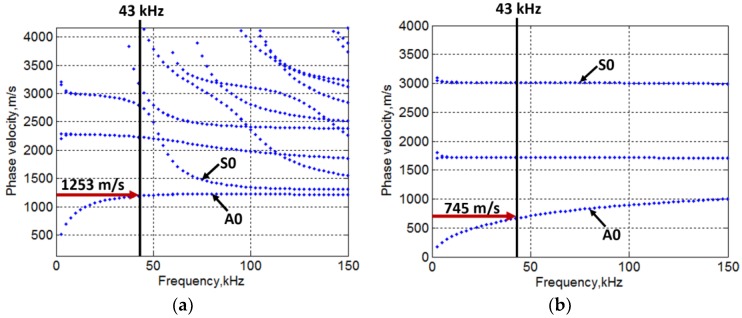
Phase velocity dispersion characteristics of defect-free (**a**) and defective region (**b**) by SAFE Method.

The semi-analytical finite element (SAFE) method [67,68,69,70] was used to calculate and plot the dispersive characteristics of multi-layered GFRP sample in the defect-free and defective regions (Figure 5a,b). The material properties and the thickness of layers used in the simulation by SAFE are illustrated in Table 2 and Table 3, respectively.

The overall thickness of defect-free and defective region selected for the simulation was 22 mm and 3.5 mm, respectively. From the SAFE simulation results (Figure 5a,b), the velocity of the A0 mode at the excitation frequency of 43 kHz was observed as 1253 m/s and 745 m/s for the defect-free and defective region, respectively.

#### 5.2.2. Application of 2D-FFT, VMD and HT

**Figure 6:**
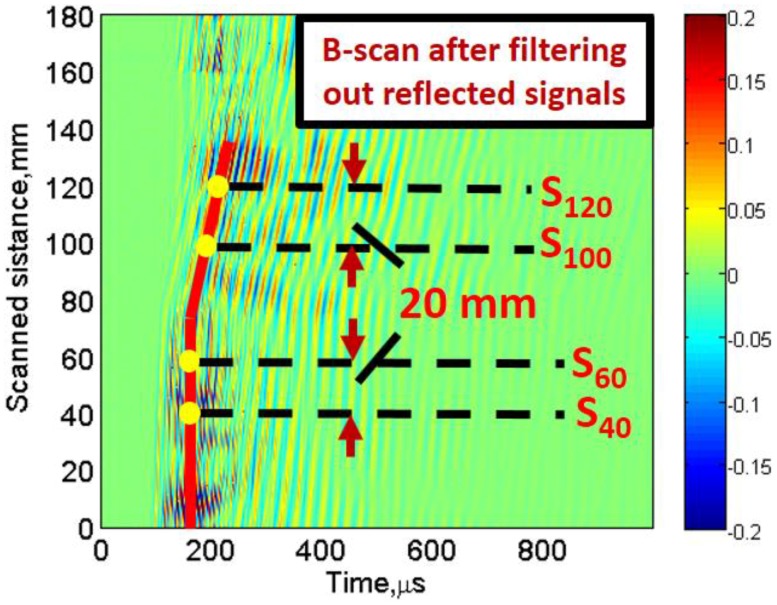
Reconstructed B-scan by considering only direct waves.

In the next step, reflected signals from the opposite edge of the sample were filtered out from the B-scan signal using 2D-FFT and reconstructed again by applying the inverse of 2D-FFT (2D-IFFT), as described in previous research [71,72]. The reconstructed B-scan with only direct waves (Figure 6) was used for the further processing.

Two A-scan signals at 40 mm (S40) and 60 mm (S60) in defect-free region and two A-scan signals at 100 mm (S100) and 120 mm (S120) at fixed distance of 20 mm were considered for calculation of approximated phase velocity and time-delays in the defect-free and defective regions, as shown in Figure 6. The VMD, as described in Section 2.3, was applied to decompose each of the four A-scans into six different IMFs to reduce the coherent noise and mode-mixing. Initially, the center frequency of each mode was considered as uniformly distributed. The typical value for the tolerance of convergence criterion was considered as 1 × 10^−7^. A maximum of 500 iterations were used in this process.

**Figure 7:**
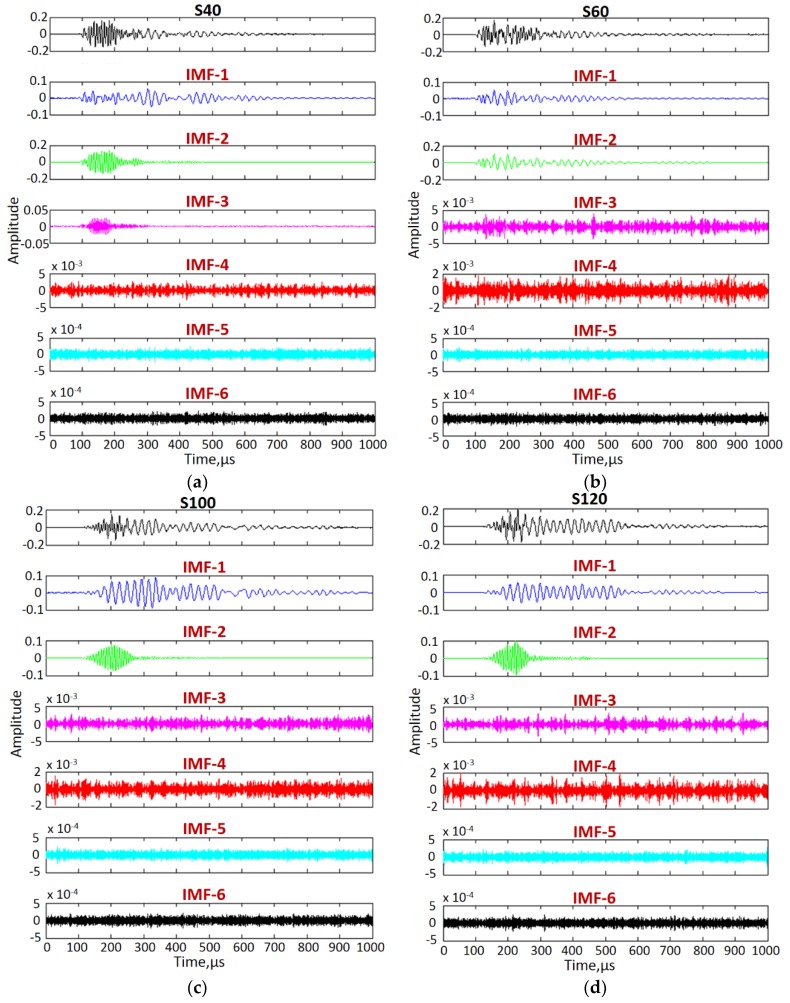
Signal decomposition using VMD: Decomposition of S40 (**a**) and S60 (**b**) defect-free signals and S100 (**c**) and S120 (**d**) defective signals into six intrinsic modes.

The A-scans (S40, S60, S100 and S120) and their intrinsic modes are presented in Figure 7a–d. The lower IMFs (IMF-3 to IMF-6) contained the significant amount of noise and hence they were removed for the reconstruction of signals. The best way to select the appropriate IMFs is by comparing the power spectral densities of all IMFs to their source signal. It was observed that power spectral densities of IMF-1 and IMF-2 in all four cases were closer to their original signals. Hence, IMF-1 and IMF-2 of all four signals were added to reconstruct new signals (S40’, S60’, S100’ and S120’).

**Figure 8:**
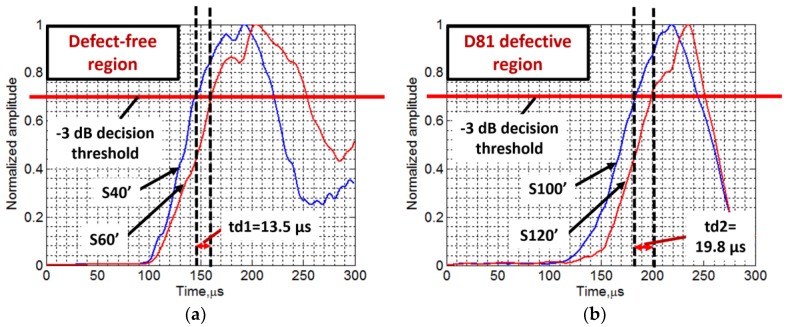
Comparison of instantaneous amplitudes of reconstructed signals: S40’ and S60’ of the defect-free region (**a**); and S100’ and S120’ of the D81 defective region (**b**).

The variation of instantaneous amplitudes of the reconstructed signals (S40’, S60’, S100’ and S120’) with time were estimated using HT, as described in Section 2.4. The time delay between the two defect-free signals (S40’ and S60’) within 20 mm distance was estimated by comparing the instantaneous amplitude characteristics (Figure 8a). Similarly, the time-delay between two signals in the defective region (S100’ and S120’) was estimated (Figure 8b). In both cases, −3 dB threshold level was used to make a decision. The time-delay (td1) between arrival times of two signals acquired at two points (spatial distance was 20 mm) apart in defect-free region was estimated as 13.5 µs, whereas the time-delay (td2) between arrival times of two signals in defective region was estimated as 19.8 µs for the same spatial distance (20 mm). Hence, the approximated value of phase velocity of the propagating wave in the defect-free region and D81 defective region was calculated as 1482 m/s and 1010 m/s, respectively (velocity = distance/time) as compared to the 1253 and 745 m/s, respectively, measured by a SAFE method. The reasons behind such differences is discussed in Section 5.2.1.

The arrival time of defect-free signals (S40’ and S60’) was observed as 146.5 µs and 160 µs, respectively, and, in the case of defective signals (S100’ and S120’) it was observed as 181.7 µs and 201.5 µs, respectively. Hence, the average time delay between the defect-free and defective signals is 38.35 µs.

## 6. Validation of the Signal Processing Approach by Investigating Another Defect

To validate the signal processing approach, a disbond type defect of 51 mm diameter located on the same GFRP sample shown in Figure 1 was inspected. A pair of contact-type transducers system (transmitter–receiver) fixed on a moving panel was used for continuously scanning up to 200 mm with a step size of 1 mm. During the scanning procedure, a transmitting transducer was excited by 100 kHz, three-period signal. Therefore, 200 A-scans were recorded with a sampling frequency of 100 MHz. Both transducers were separated by 50 mm optimal distance and operated in pitch-catch mode. It should be noted that optimal distance of 50 mm between two contact-type transducers during the experimental investigation was selected in such a way that it should approximately be equal to the few wavelengths of slowest A0 mode, which also ensured maintaining the desired resolution of transmitting and receiving GWs during interaction with the object [26,73]. The significant amount of changes in the received waveform of GW would suggest the possible presence of defects in the sample.

**Figure 9:**
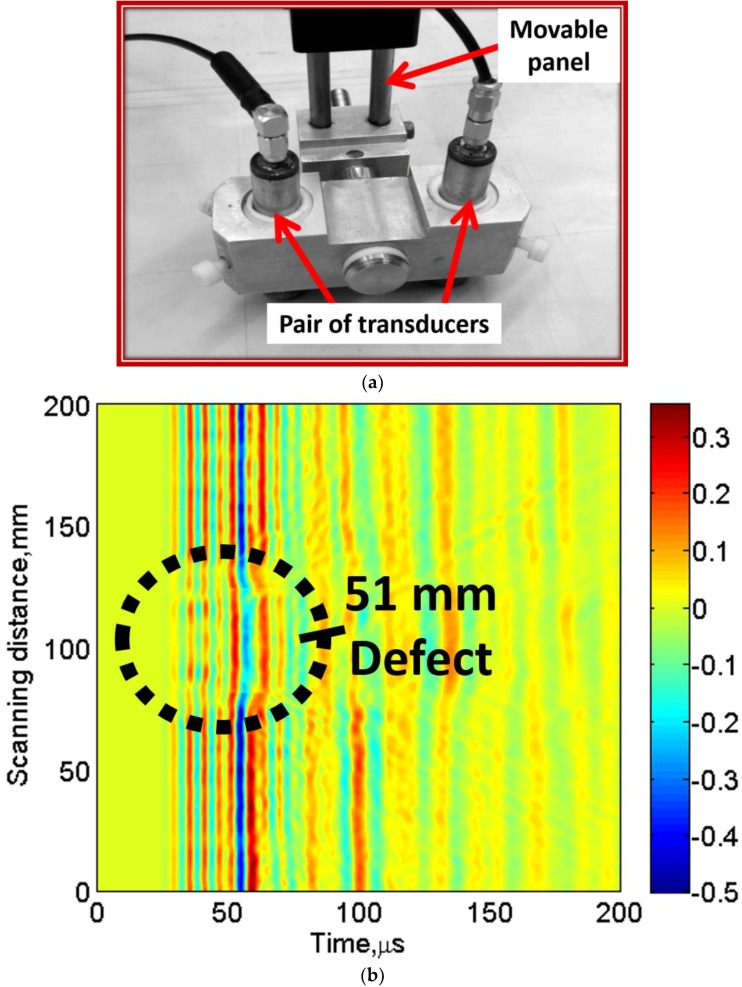
Experimental investigation of 51 mm defect located on GFRP sample: (**a**) photo view of special arrangement of contact-type transmitter–receiver pair mounted on a moving panel; (**b**) experimental B-scan showing 51 mm defect.

The characteristics of the transducers and LF ultrasonic system used in the experiment are specified in Section 3.1. The initial position of the defect from the initial point of the scanning was 83 mm. The photo-view of the transducer system and the acquired B-scan are presented in Figure 9a,b, respectively.

**Figure 10:**
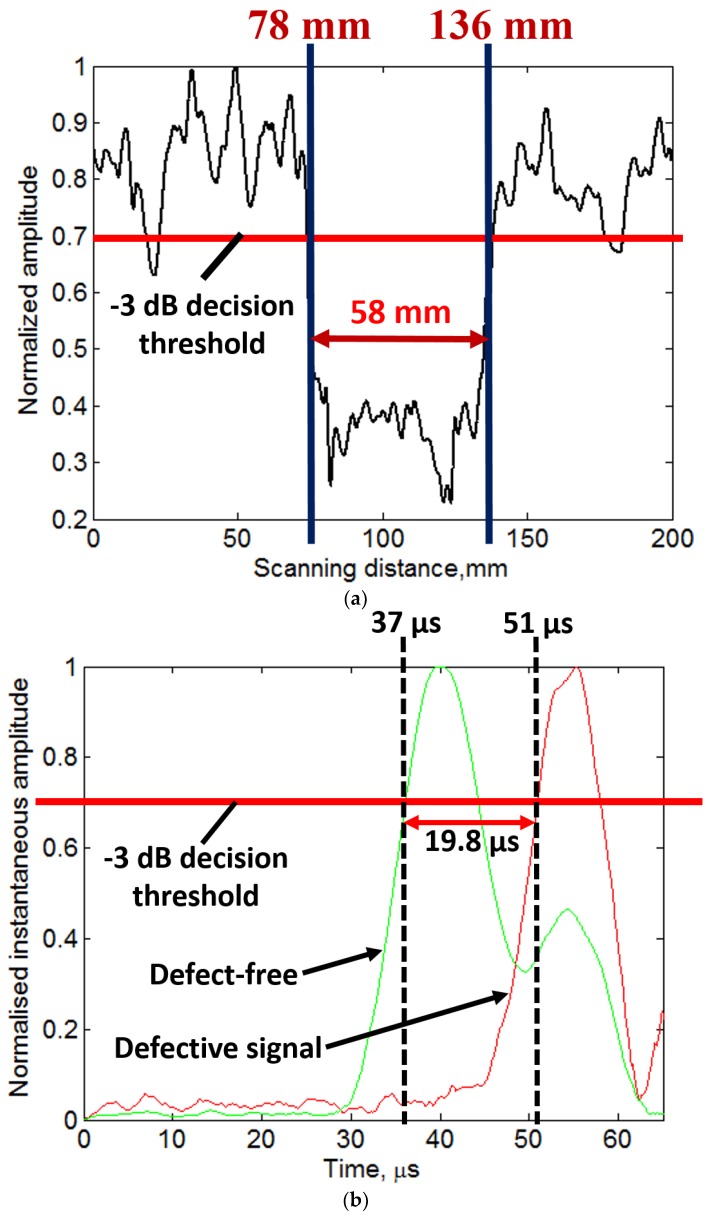
Signal processing to investigate 51 mm defect: (**a**) amplitude detection after wavelet denoising; and (**b**) comparison of instantaneous amplitudes of defect-free and 51 mm defective signal.

In the next step, the signal processing approach presented in Section 4 and Section 5 were applied on the experimental B-scan (Figure 9b). Therefore, DWT, 2D-FFT, VMD and HT were used to extract the defect features. The results to estimate the size and location and the phase velocities of propagating wave modes are shown in Figure 10a,b. The results of amplitude detection presented in Figure 10a show that the 51 mm defect is observed as 58 mm (percentage error is 12%) and the location of defect from the initial scanned point is observed as 78 mm (percentage error is 3.6%). The instantaneous amplitudes of defective signal (at 100 mm) and defect-free signal (at 25 mm) are compared (Figure 10b) after VMD. By applying the −3 dB threshold, the arrival time of propagating wave is observed as 37 µs for the defect-free region and 51.5 µs in the case of 51 mm defective region. As the distance of transmitter and receiver is fixed, the velocity of propagating wave is 1351 m/s and 981 m/s in the defect-free and defective region, respectively. These values are closer to the phase velocities of A0 waves estimated by SAFE method (1254 m/s for defect-free and 900 m/s for defective region at 100 kHz frequency).

The phase velocities in these cases are lower as compared to the previous experiment based on MFC and contact-type transducer. Therefore, irrespective of other factors such as thickness and type of materials, the accuracy in results also depends on the type of transducers and the excitation frequency. The results of both experiments lead to conclude that presented signal processing algorithm is quite effective, especially for investigating the disbond type defects using contact-type transducers. The limitations associated with this approach are discussed in Section 7.

## 7. Limitation and Issues of the Presented Technique

The accuracy of the results depends on the limitations associated with the experimental method, environmental and operating conditions and the amplitude-based signal processing approaches. The linear ultrasonic method used here for the experimental investigation is only applicable to the analysis of large defects or defects with diameters greater than the operating wavelength. Therefore, the presented experimental method is not suitable to detect and locate micro-cracks. To detect micro-cracks in the structure, non-linear scanning techniques [74,75] can be used, which are highly sensitive to micro-cracks as compared to linear ultrasonic methods. The peridynamic theory can also be an alternative to develop a numerical model for detection of micro-cracks during early stage [76]. However, the validation of this method is required. It should be noted that wave phenomena such as scattering, mode conversions and reflections are not considered in SAFE method. However, in the real sample, as the GW travels from defect-free to defective region, these wave phenomena may arise.

The amplitude-based processing approach is very sensitive to the operating temperature. Moreover, the thickness and properties of the conducting glue can be significantly changed with the temperature variations. Hence, the room temperature of 25 °C has been maintained throughout the investigation. Although DWT is used to denoise the experimental signals, the amplitude variations sometimes cannot give the appropriate results to locate and size the defects. As the receiving transducer is moved away from the MFC transmitter in the experimental approach-1 (Section 3), the effect of signal attenuation may also be considered. Moreover, the signal processing algorithm is tested only on the experimental signals acquired by contact-type transducers operating in thickness mode which are more sensitive *to out-of-plane* displacements. The reproducibility of results depends on the difference in amplitude variations in the defective and defect-free regions.

During the experimental investigation of a 51 mm defect as described in Section 6, the fixed distance of 50 mm was selected between the transducers for continuous scanning up to the scanning distance of 200 mm. However, it created the unknown time-offset. Moreover, the accuracy of results also depends on the decision threshold (−6 dB, −3 dB or a maximum of the peak) applied to compare the instantaneous amplitudes. The results can be improved by taking two separations between the transducers which can cancel out the unknown offsets.

## 8. Conclusions

This work demonstrates the implementation and validation of signal processing algorithm to increase the accuracy in the measurement of size and location of the disbond-type defects located on the GFRP sample by processing a single B-scan. Moreover, the method based on 2D-FFT, VMD and HT for the estimation of phase velocities of the propagating waves in the defective and defect-free region of the sample is also proposed without using the dispersion curves.

The work can be summarized as follows:

- Two experiments using LF ultrasonic system were performed for the analysis of disbond-type defects by GWs. In the first experiment, P1-type MFC transducer (transmitter) was glued on the sample and contact-type piezoceramic transducer (receiver) was scanned up to 180 mm to investigate the defect of 81 mm diameter. In the second experiment, two contact-type transducers fixed on a moving panel were used to investigate the defect of 51 mm diameter by continuous scanning up to 200 mm. The defects are marginally detectable in the B-scans.
- The DWT along with amplitude detection technique was applied on experimental B-scans to locate and size the defects with a significant accuracy (percentage error was less than 12%).
- By combining the features of 2D-FFT, VMD and HT, the phase velocities and time-delays of the propagating waves in defective and defect-free regions were calculated and compared with the SAFE method. The results show good accuracy despite the variable thickness of the sample.

It should be noted that major factor in terms of accuracy in the measurement of phase velocities in the presented approach is the selection of threshold decision level (−3 dB, −6 dB or maximum of peak) to compare the instantaneous amplitudes of the signals. Moreover, the future scope is to develop the signal processing algorithm based on other parametric estimations other than amplitudes. The improvement in this work can also be performed by rectifying the discussed limitations associated in the presented approach.

## Figures and Tables

**Table 1:** Parametric specifications of LF ultrasonic system.

Parameters	Numerical Value
No. of input channels	2
No. of bits of analog-to-digital converter	10
Digitization rate	100 MHz
Overall system gain (maximum)	113 dB
Resolution of mechanical scanner	20 µm
Ultrasonic system to computer interface	USB V.2

**Table 2:** GFRP material properties.

Parameters	Numerical Value
**Paint (Surface layer):**	
Density (*ρ*)	1270 kg/m^3^
Young’s modulus (E)	4.2 GPa
Poisson’s ratio (υ)	0.35
**Unidirectional GFRP layer:**	
Density (ρ)	1828 kg/m^3^
Young’s modulus (E1)	42.5 GPa
Young’s modulus (E2)	10 GPa
Poisson’s ratio (υ12)	0.26
Poisson’s ratio (υ23)	0.4
In plane shear modulus (G12)	4.3 GPa
**Epoxy:**	
Density (ρ)	1260 kg/m^3^
Young’s modulus (E)	3.6 GPa
Poisson’s ratio (υ)	0.35

**Table 3:** The thickness of layers in defect-free and defective region.

Parameters	Numerical Value
**Defect-free region:**	
Thickness of paint	0.5 mm
GFRP (0°/90°/45°/−45°/0°) layer	2 mm
Epoxy	1 mm
GFRP (45°/−45°) layer	18.5 mm
**Defective region:**	
Thickness of paint	0.5 mm
GFRP (0°/90°/45°/−45°/0°) layer	2 mm
Epoxy	1 mm

## Nomenclature

*GFRP*	Glass fiber reinforced plastic
*CFRP*	Carbon fiber reinforced plastic
*NDT*	Non-destructive testing
*GW*	Guided wave
*SHM*	Structural health monitoring
*WTB*	Wind turbine blade
*DWT*	Discrete wavelet transform
2D-*FFT*	Two-dimensional fast Fourier transform
*HT*	Hilbert transform
*VMD*	Variational mode decomposition
*EMD*	Empirical mode decomposition
*EEMD*	Ensemble empirical mode decomposition
*SSP*	Split-spectrum signal processing
*LF*	Low-frequency
*LPF*	Low-pass filter
*HPF*	High-pass filter
*db*	Daubechies
*x*(*n*)	Original signal to be processed
*h_h_* (*n*)	Filter function of *HPF*
*h_l_* (*n*)	Filter function of *LPF*
*Y_h_* [*k*]	Response of *HPF*
*Y_l_* [*k*]	Response of *LPF*
*M*	Maximum level of decomposition
*N*	Length of signal *x*(n)
σ _j_^noise^	Estimated noise level in DWT
*x*(*t*)	Real-valued signal
*X_h_* (*t*)	Hilbert transform of x(*t*)
*x_a_* (*t*)	Analytical signal of *x*(*t*)
*A_i_* (*t*)	Instantaneous amplitude
*Φ* (*t*)	Instantaneous phase
*fi* (*t*)	Instantaneous frequency
*H*	Transfer function in 2D-FFT
*u_k_*	Set of all modes for a real-valued signal in VMD
*δ*	Dirac distribution
*S*0	Fundamental symmetric mode
*A*0	Fundamental asymmetric mode
